# Testing spasticity mechanisms in chronic stroke before and after intervention with contralesional motor cortex 1 Hz rTMS and physiotherapy

**DOI:** 10.1186/s12984-023-01275-9

**Published:** 2023-11-08

**Authors:** Wala Mahmoud, Hans Hultborn, Jagoba Zuluaga, Christoph Zrenner, Brigitte Zrenner, Ulf Ziemann, Ander Ramos-Murguialday

**Affiliations:** 1 Institute for Clinical Psychology and Behavioral Neurobiology, University of Tübingen, Tübingen, Germany; 2 Department of Neurology & Stroke, University of Tübingen, Tübingen, Germany; 3 Hertie Institute for Clinical Brain Research, University of Tübingen, Eberhard Karls University Tübingen, Hoppe-Seyler-Straße 3, 72076 Tübingen, Germany; 4 Department of Neuroscience, University of Copenhagen, Copenhagen, Denmark; 5 Tecnalia, Basque Research and Technology Alliance, San Sebastián, Spain; 6 Athenea Neuroclinics, San Sebastián, Spain

**Keywords:** Stroke, Spasticity, rTMS, Spinal circuitry, Stretch reflex, Passive stiffness

## Abstract

**Background:**

Previous studies showed that repetitive transcranial magnetic stimulation (rTMS) reduces spasticity after stroke. However, clinical assessments like the modified Ashworth scale, cannot discriminate stretch reflex-mediated stiffness (spasticity) from passive stiffness components of resistance to muscle stretch. The mechanisms through which rTMS might influence spasticity are also not understood.

**Methods:**

We measured the effects of contralesional motor cortex 1 Hz rTMS (1200 pulses + 50 min physiotherapy: 3×/week, for 4–6 weeks) on spasticity of the wrist flexor muscles in 54 chronic stroke patients using a hand-held dynamometer for objective quantification of the stretch reflex response. In addition, we measured the excitability of three spinal mechanisms thought to be related to post-stroke spasticity: post-activation depression, presynaptic inhibition and reciprocal inhibition before and after the intervention. Effects on motor impairment and function were also assessed using standardized stroke-specific clinical scales.

**Results:**

The stretch reflex-mediated torque in the wrist flexors was significantly reduced after the intervention, while no change was detected in the passive stiffness. Additionally, there was a significant improvement in the clinical tests of motor impairment and function. There were no significant changes in the excitability of any of the measured spinal mechanisms.

**Conclusions:**

We demonstrated that contralesional motor cortex 1 Hz rTMS and physiotherapy can reduce the stretch reflex-mediated component of resistance to muscle stretch without affecting passive stiffness in chronic stroke. The specific physiological mechanisms driving this spasticity reduction remain unresolved, as no changes were observed in the excitability of the investigated spinal mechanisms.

**Supplementary Information:**

The online version contains supplementary material available at 10.1186/s12984-023-01275-9.

## Background

Besides paresis, post-stroke disability often arises from spasticity and soft tissue contractures which emerge weeks or months after the injury. Spasticity, which impacts 20–40% of survivors [1] can contribute to issues such as restricted range of motion (ROM), abnormal posture, and pain [2]. Lance's widely cited 1985 definition describes spasticity as a “velocity-dependent increase in tonic stretch reflexes (muscle tone) with exaggerated tendon jerks, resulting from hyperexcitability of the stretch reflex as part of the upper motor neuron syndrome” [3].

Many non-invasive and non-pharmacologic intervention options are emerging for treatment of spasticity [4, 5]. In particular, there is growing evidence to support the use of the non-invasive brain stimulation method, repetitive transcranial magnetic stimulation (rTMS), for reducing spasticity after stroke [6–12]. RTMS elicits an electric field within the brain, causing alterations in the excitability of neurons not only in the targeted brain areas at the stimulation site but also in distant brain areas, including the contralateral motor cortex and subcortical structures [13–15]. The precise mechanism by which rTMS might cause behavioral effects on spasticity is unclear. It is plausible that rTMS can modulate the activity of the spinal circuitry, implicated in spasticity observed in spastic stroke patients, by modifying the excitability of cortical centers that project to this circuitry [16–18].

A recent systematic review of randomized controlled trials concluded that the use of contralesional motor cortex 1 Hz rTMS has a positive effect on reducing spasticity as estimated from the modified Ashworth scale (MAS) [19]. The MAS [20] is performed by passively stretching a joint and simultaneously estimating the perceived resistance on a 6-point ordinal scale. Though widely used, the MAS suffers from poor reliability, sensitivity and objectivity [2, 21–23]. Importantly, the examiner perceiving the resistance cannot discriminate the velocity-dependent stretch reflex, i.e., true spasticity, from the passive stiffness that results from changes that occur in the muscle and the surrounding soft tissues after the injury [24–29]. Establishing therapeutic effects of rTMS on spasticity requires careful quantification of the reflex-mediated component of the resistance to muscle stretch and discriminating it from the passive stiffness components.

Significant progress has been achieved in recent years regarding the development and testing of devices that enable the objective quantification of spasticity and discrimination of the different components which contribute to the resistance to passive joint stretch [25, 30–34]. Using a hand-held dynamometer, which enables the simultaneous recording of biomechanical and muscle activity data [29, 33–37], we objectively measured the stretch reflex-mediated stiffness in the wrist joint in a cohort of chronic stroke patients [38].

Central to the pathophysiology of spasticity is the excitability of the monosynaptic Ia afferent-motoneurone (MN) pathway underlying the stretch reflex [39]. The excitability of the stretch reflex circuit is regulated by complex spinal circuitries, which themselves are modulated by supraspinal pathways descending from cortical and brainstem structures [40]. After an upper motor neuron injury, there is an imbalance in the cortical and subcortical regulatory input to the spinal cord, which triggers secondary changes in the excitability of the spinal circuitry over weeks and months [41–43]. As a result, reflex hyperexcitability emerges as a gradual adaptation in the spinal circuitry distal to the lesion [42, 44–46]. Changes in the excitability of certain pathways and their contribution to the clinical picture of spasticity has been the topic of many studies in humans and animal models in the last decades [40, 44, 47–49].

Multiple spinal inhibitory mechanisms have been found to be reduced in spastic stroke patients and identified as potentially contributing to stretch reflex hyperexcitability in both upper and lower limbs (for review see [44, 46]). These include (1) post-activation depression, a frequency-dependent reduction in the release of neurotransmitters from previously activated fibers. A mechanism that has consistently been found to be reduced on the affected but not the unaffected side in spastic patients after stroke, both in the lower [50–52] and upper limbs [50, 53]. The extent of the reduction in post-activation depression was also found to be related to the degree of spasticity measured clinically in the lower limb [50, 52] as well as in the upper limb [53]; (2) presynaptic inhibition of Ia terminals, a mechanism which modulates the synaptic transmission from Ia afferents before they reach the target neurons. Multiple studies have reported a significant reduction in presynaptic inhibition in the upper limb in the stroke population [54–56], but this reduction was found to be not exclusive to the affected side [53]; (3) reciprocal inhibition from muscle spindles of the antagonist muscle group. This disynaptic inhibition is mediated through Ia afferents and Ia inhibitory interneurons, which are normally controlled by excitatory descending pathways including the corticospinal tract [46]. At rest, a decrease in Ia reciprocal inhibition has been observed in spastic patients in the upper limb [55, 56] and even potentially converted into facilitation from flexors to extensors in the lower limb [57–59].

An intervention which causes clinical improvements in “spasticity” would be expected to interact with its central pathophysiological mechanisms i.e., the excitability of the stretch reflex and the spinal circuits involved in its modulation. In humans, corticospinal neurons project to a large group of spinal interneurons and modulate their activity [60–64]. It is likely that rTMS can change the excitability of the spinal circuitry by modulating the excitability of cortical centers that project to this circuitry [16–18]. In addition, rTMS can modify transmission in neuronal circuitries in deeper lying structures in the brain including the brain stem, which itself plays an important role in controlling the spinal reflex excitability through direct and indirect projections [40].

In this study we aimed to objectively quantify the effects of an rTMS and physiotherapy intervention on spasticity of the wrist flexors in chronic stroke patients. Additionally, we explored possible mechanisms through which the cortical effects of rTMS might interact with spinal mechanisms thought to be related to stretch reflex hyperexcitability.

## Methods

### Subjects

Fifty-four chronic stroke patients (38 males, 16 females) with an average age of 58 ± 12 years participated in this study. All patients had unilateral hemiparesis due to stroke at least 6 months prior to inclusion (46 ± 42 months). The study was conducted at the TMS outpatient clinic, Department of Neurology, Tübingen University Hospital in Germany between September 2019 and March 2022.

Before enrolling in the study, a neurologist thoroughly examined the patients and determined their suitability for receiving rTMS intervention based on the clinical safety guidelines put forward by Rossi et al. [65]. The criteria for receiving rTMS included: (1) no seizures, or a seizure-free period of at least 4 months prior to the commencement of the therapy; (2) for patients with intracranial implants (e.g., aneurysm clips, shunts, stimulators), a minimum distance of 8 cm between the TMS coil and the implant. Because this study is primarily concerned with examining the physiological changes which relate to rTMS intervention, patients were included in the study even when they did not exhibit any spasticity at the wrist. Patients who suffered from severe contractures in the wrist joint which impeded the placement of the hand in the orthosis (n = 3) were not measured using the hand-held dynamometer. Detailed patient characteristics are provided in Table 1. After the clinical screening, anatomical T1-weighted magnetic resonance imaging (MRI) was acquired for MRI-guided TMS neuronavigation.

**Table 1:** Characteristics of study participants

ID	Age (years)	Sex	Time since stroke (months)	Affected side	Ischemic /hemorrhagic	Location	FMA-UE/66	MAS wrist	MAS total/70	Visual analog scale (%)	MAL (%)	Disability rating scale/24
2	66	Female	92	Left	Ischemic	Middle cerebral artery	6	2	17	50	0	12
3	76	Female	16	Left	Ischemic	Middle cerebral artery	34	1+	12	15	8	8
4	46	Male	16	Left	Hemorrhagic	Right frontal intracranial bleeding	8	0	7	10	0	14
6	70	Female	240	Left	Ischemic	Middle cerebral artery	8	4	24	45	0	9
8	44	Female	23	Left	Hemorrhagic	Basal ganglia	41	2	12	50	25	17
9	65	Male	25	Left	Ischemic	Thalamus	7	3	35	70	0	11
12	52	Female	14	Left	Ischemic	Anterior cerebral artery	4	0	3,5		0	
13	84	Male	104	Left	Ischemic	Middle cerebral artery (lenticulostriate)	8	1+	8,5	40	2	15
14	71	Female	63	Right	Hemorrhagic	Frontoparietal subdural hematoma	11	4	28	100	0	22
15	55	Male	38	Right	Ischemic	Middle cerebral artery	13	1	8,5		0	18
16	54	Male	14	Left	Hemorrhagic	Basal ganglia	12	3	23	50	6	16
18	55	Female	18	Left	Hemorrhagic	Basal ganglia	39	0		0	38	7
19	54	Male	51	Right	Ischemic	Medulla oblongata/cerebellum	55	1+	5	51	6	19
20	67	Male	46	Left	Hemorrhagic	Basal ganglia	19	1+	7	50	0	15
21	51	Male	9	Left	Ischemic	Middle cerebral artery	7	1+	7,5	50	0	15
22	53	Female	22	Right	Hemorrhagic	Frontoparietal intracranial bleeding	42	0	8,5	80	7	9
23	54	Male	32	Right	Ischemic	Anterior cerebral artery	5	1	9,5	50	0	12
26	54	Male	37	Left	Ischemic	Anterior choroidal artery	10	0	8	50	0	21
27	58	Male	35	Left	Ischemic	Basal ganglia	10	1+	6	0	0	18
28	74	Male	14	Right	Ischemic	Middle cerebral artery	18	2	10,5	60	23	14
29	38	Male	13	Left	Hemorrhagic	Basal ganglia	14	1	6,5	30	3	
31	51	Male	48	Left	Hemorrhagic	Basal ganglia	26	0	15,5	75	6	13
32	44	Male	32	Left	Ischemic	Middle cerebral artery	11	2	14,5	30	0	3
33	57	Female	20	Left	Ischemic	Middle cerebral artery	41	0	6,5	0	10	11
34	36	Male	64	Right	ischemic	Middle cerebral artery	28	0	9,5		0	14
35	73	Male	8	Left	ischemic	Middle cerebral artery	38	1	7	15	9	10
36	64	Male	45	Left	Hemorrhagic	Left intracranial bleeding	14	0	1	25	0	11
37	60	Male	19	Left	Ischemic	Middle cerebral artery	16	0	10	60	0	12
38	29	Female	27	Right	Hemorrhagic	Left intracranial bleeding	14	1+	12	80	0	11
39	48	Male	88	Left	Hemorrhagic	Basal ganglia	9	3	21	100	0	21
40	56	Male	37	Left	Ischemic	Middle cerebral artery	16	4	29,5	80	3	17
41	27	Male	17	Left	Hemorrhagic	Right intracranial bleeding	18	2	13,5	60	6	
42	52	Male	39	Right	Ischemic	Middle cerebral artery	48	1+	5	25	28	6
43	70	Male	43	Right	Hemorrhagic	Basal ganglia	46	1+	7,5	50	9	12
44	70	Male	95	Right	Hemorrhagic	Left intracranial bleeding	7	2	21,5	50	0	13
46	77	Female	43	Left	Ischemic	Right internal carotid	4	4	23		0	
47	58	Female	80	Left	Ischemic	Middle cerebral artery	43	2	17	45	44	5
48	71	male	40	Left	Ischemic	Cerebral peduncle	47	1+	13,5	35	19	11
49	64	Female	6	Left	Ischemic	Middle cerebral artery	32	2	14,5	50	0	12
1001	70	Female	40	Right	Ischemic	Middle cerebral artery	10	1	6,5	20	0	8
1004	62	Male	16	Right	Hemorrhagic	Basal ganglia	22	1+	6,5	15	0	13
1007	69	Male	120	Right	Ischemic	Anterior &middle carotid arteries	39	1+	14	60	5	20
1009	54	Male	179	Left	Ischemic	Middle cerebral artery	58	1	4	40	35	8
1014	53	Male	45	Left	Ischemic	Posterior cerebral artery	31	1	11	50	10	10
1018	70	Male	36	Left	Ischemic	Frontoparietal	57	0	6	40	52	5
1023	54	male	47	Right	Hemorrhagic	Thalamus	28	2	18,5	60	43	7
1024	67	Male	50	Left	Ischemic	Basal ganglia	32	3	16,5	100	26	16
1039	49	Male	73	Right	Ischemic	Middle cerebral artery	38	3	17	75	14	4
1044	73	Male	71	Left	Ischemic	Middle cerebral artery	42	1+	9	30	21	11
1055	58	Male	27	Right			56	0	0	0	30	7
1061	66	Male	36	Right	Ischemic	Middle cerebral artery	34	1	10	55	18	12
1065	52	Male	29	Left	Ischemic	Middle cerebral artery	45	1	9	50	16	16
1076	44	Female	12	Right	Ischemic	Middle cerebral artery	59	0	6,5	0	69	4
1077	59	Female	29	Right	Hemorrhagic	Left intracranial bleeding	21	2	15,5	30	0	20

The clinical scores represent those acquired before the intervention. FMA-UE: Fugl-Meyer Assessment-Upper Extremity; MAS: modified Ashworth scale; MAL: motor activity log, a structured interview which examines the patient’s use of the affected upper limb in comparison to the time before the injury

### Intervention paradigm

To determine optimal stimulation sites and coil orientation during the therapy, we first mapped the area surrounding the precentral gyrus of the motor cortex in the non-lesioned hemisphere utilizing the patient MRI images integrated within the neuronavigation system (Nexstim, Helsinki). Biphasic single pulse TMS (with a posterior anterior initial phase) was used to identify the motor hotspot for the abductor pollicis brevis (APB) and first dorsal interosseous (FDI) muscles. Surface EMG recordings were acquired using bipolar adhesive electrodes (Ambu® Neuroline 720, Copenhagen) placed on the bellies of APB and FDI muscles, with a reference electrode on the ulnar styloid process. TMS was delivered using a 72 mm figure-of-eight coil figure-of-eight coil (Nexstim Focal Coil, Nexstim, Helsinki) placed perpendicular to the motor cortex. Surface EMG recordings were acquired using bipolar adhesive electrodes (Ambu® Neuroline 720, Copenhagen) placed on the bellies of APB and FDI muscles, with a reference electrode on the ulnar styloid process. TMS was delivered using a 72 mm figure-of-eight coil figure-of-eight coil (Nexstim) placed perpendicular to the motor cortex. The motor hot spot was defined as the location which elicited the largest motor-evoked potentials (MEPs) in APB or FDI. The resting motor threshold (RMT) was defined as the lowest stimulation intensity which triggered an MEP with a peak-to-peak amplitude of at least 50 µV in at least 5 out of 10 consecutive stimulations delivered to the motor hot spot. The muscle with lower RMT served as the reference for the therapy. The neuronavigation system with head and TMS coil trackers, along with a stereotactic camera enabled precise registration of the TMS coil's location relative to the patient's head and specific brain area.

During therapeutic rTMS, subjects were seated in an electronically adjustable reclining chair with relaxed arms. The navigated brain stimulation system delivered 1200 biphasic pulses at 1 Hz to the motor hot spot in the contralesional motor cortex at a stimulation intensity of 120% of the reference muscle's RMT utilizing the same coil location and orientation as registered during the mapping session. The use of the neuronavigation system enabled the consistent targeting of the initial hot spot throughout the intervention. Each subject received 3 rTMS sessions per week for a duration of 4–6 weeks (~ 15 sessions). A single rTMS session lasted ≈30 min including 10 min of preparation and 20 min of rTMS.

In order to enhance the effects of rTMS for motor rehabilitation purposes, patients received 50 min of personalized exercise-based physiotherapy immediately following the end of the rTMS session. The training focused on the arm and hand function including mobility exercises, strength training, object manipulation and fine motor training.

### Evaluation sessions

A battery of assessments took place before the start and at the end of the intervention. These included (a) clinical evaluation of spasticity, motor impairment and motor function, as well as patient-centered outcome measures; (b) objective evaluation of spasticity using a hand-held dynamometer; (c) electrophysiological measurement of three spinal circuit mechanisms. All measurements were performed by a single experimenter who is an experienced researcher and physiotherapist (first author).

- Clinical tests

The clinical tests included the Fugl-Meyer Assessment scale-upper extremity (FMA-UE), the Wolf motor function test (WMFT), and the modified Ashworth scale (MAS).

The FMA-UE [66] is a quantitative measure of upper limb impairment in stroke patients. It includes 33 performance-based items that are rated on a 3-point ordinal scale (0 cannot perform, 1 can perform partially, and 2 can perform fully) with a maximum total score of 66 points.

The Wolf Motor Function test (WMFT) [67] includes 15 timed tasks for the evaluation of upper limb motor function. Both the duration (in seconds) required to perform each task as well as the quality of the movement are recorded. Movement quality is measured on an ordinal scale between 0 and 5 (0: no movement attempt, 1: movement attempt failed, 2: minimal synergy, 3: significant synergy, 4: almost normal, 5: movement is normal). The total time score is the sum of the time required for each task (when the task is not completed within 120 s, the performance time is recorded as 120 s), and the quality score is the sum of quality scores for all the tasks, with a maximum score of 75.

To estimate the MAS score in the wrist extensors, the examiner moved the wrist joint from full flexion to full extension and simultaneously estimated the perceived resistance according to a 6-point ordinal scale (0: no increase in muscle tone, 1: slight increase in muscle tone at the end of the ROM, 1+: slight increase in muscle tone throughout less than half of the ROM, 2: marked increase in muscle tone in most of the ROM, 3: passive movement difficult, 4: affected parts rigid). To get a total MAS score for the upper limb, we repeated the same measurement procedure for multiple joints and added the individual MAS scores of 14 movements in these joints (flexion, abduction, internal rotation and external rotation; of the shoulder; flexion and extension of the elbow; pronation and supination of the forearm; flexion and extension of the wrist; flexion and extension of the fingers; and adduction and abduction of the thumb). The score 1 + was transformed into 1.5 to allow mathematical addition of the scores.

Three questionnaires were used to capture the subjective experience of the patients: (1) *visual analogue scale (VAS)* where patients reported the degree of spasticity they perceived in their arm on a scale that ranged between 0 and 100, where 0 is no spasticity at all and 100 is the highest spasticity imaginable; (2) *Motor Activity Log (MAL-30)*, a structured interview where patients described the frequency with which they used the affected limb to perform each of 30 different activities of daily living [68, 69]. The scores range between 0 and 5 where 0 is never and 5 is exactly as much as before the injury. The reason why the patient did not use the affected limb for a particular task was also noted. Whenever the reason was related to side dominance (e.g., writing with the left affected side when the patient is right-handed) or when the activity is irrelevant to the patient’s life (combing hair when the patient is bold), the question was discarded and the highest score for that question (5 points) was subtracted from the total score. The final score was then expressed as a ratio; (3) *Disability rating scale* [70, 71] a scale that is concerned with the effect of spasticity on activities of daily living. Patients were asked to report the difficulty they experienced in performing six activities on a scale from zero to five (0 no difficulty and 5 cannot perform the task). The activities included: palm hygiene, entering the spastic arm in a sleeve, washing the underarm, washing the elbow, cutting fingernails and doing exercises with the arm.

b. Objective assessment of spasticity in the wrist flexors using a hand-held dynamometer

**Fig. 1:**
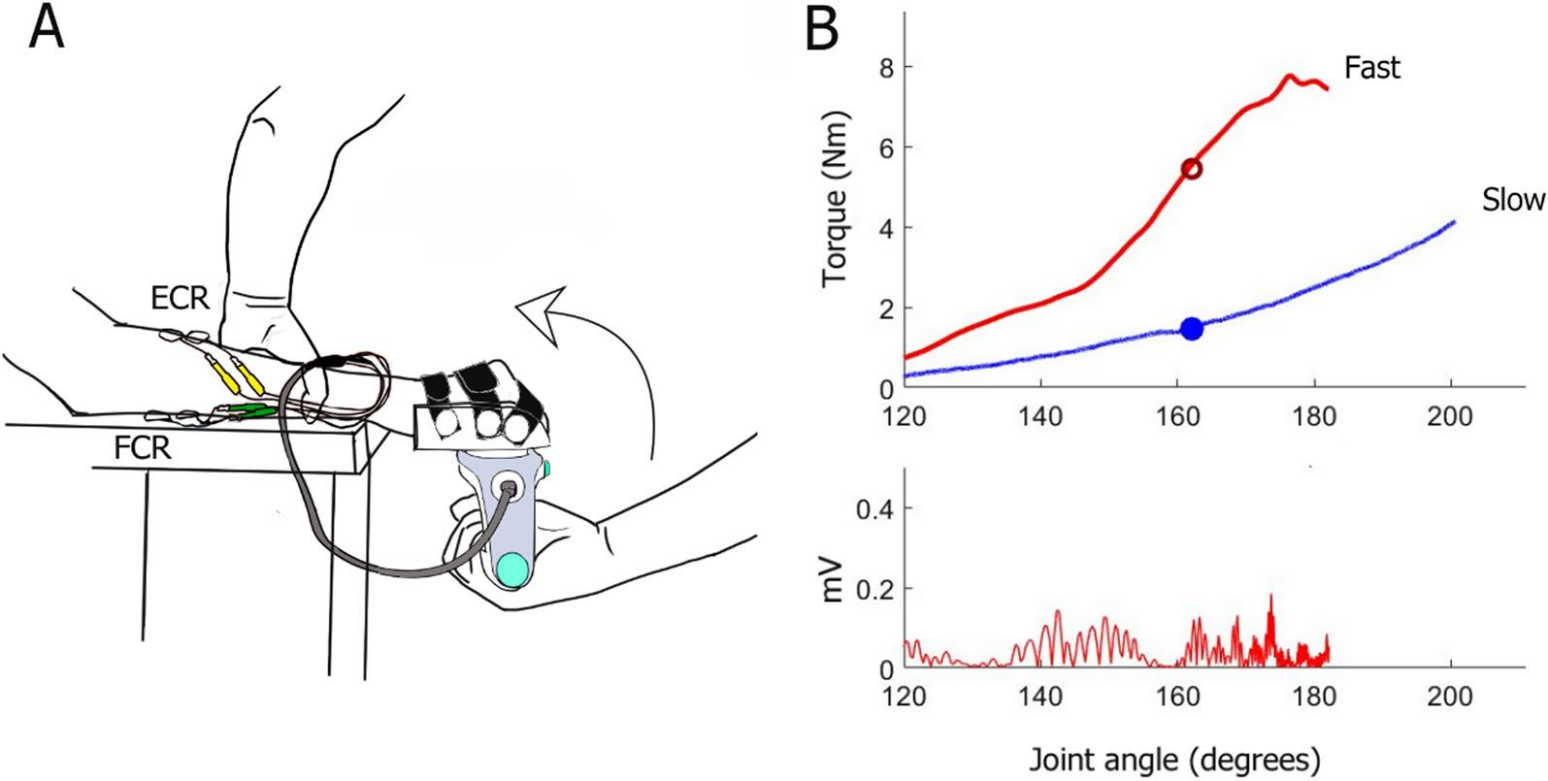
PSAD assessment set up and an example dataset. **A** The PSAD is designed as a handle, which attaches to a joint-specific orthosis, in this case: a hand orthosis. The patient's hand was comfortably fitted inside the orthosis using Velcro straps. The subject was seated in an armless chair with the investigated arm placed on a height-adjustable table. The shoulder was slightly abducted, the elbow semi-flexed and the forearm pronated. In this position, the wrist and hand extend slightly outside the edge of the table. The hand size (measured as the distance (cm) between the third knuckle and the middle of the wrist joint), orthosis size (small, medium, or large), and height and weight of the subject were also recorded and used for the optimization of the signal analysis. EMG was recorded from Flexor Carpi Radialis (FCR-green) and Extensor Carpi Radialis (ECR-yellow) muscles using bipolar surface adhesive electrodes. The experimenter moved the hand of the patient throughout the whole available wrist extension range of motion (ROM) at either a slow (< 20°/s) or a fast (> 300°/s) velocity. **B** The upper panel is an example of torque data collected during slow (blue) and fast (red) stretches. The red circle represents the point in the ROM where the stretch reflex mediated torque was obtained while the blue circle represents the corresponding point during slow trials where the passive stiffness component was obtained. The lower panel is the FCR rectified EMG data corresponding to the fast trial. Figure 1A has been modified from [38]

The assessment of resistance to wrist extension was done using the Portable Spasticity Assessment Device (PSAD) (Movotec, Denmark). The PSAD is a hand-held dynamometer which encloses multiple sensors including dynamometers, accelerometers, and a gyroscope to enable the simultaneous acquisition measurement of force, joint movement, and muscle activity [29, 33–37]. The PSAD has been validated for measurement of passive and reflex-mediated stiffness in the ankle joint plantar flexors in previous studies. We have demonstrated validity and reliability of the device for quantifying the reflex-mediated stiffness in the wrist flexors in chronic stroke patients [38] (Fig. 1).

To assess the passive and reflex-mediated components of the resistance to wrist extension, the experimenter moved the wrist joint throughout the available range of motion by applying force through the handle of the device at slow and fast velocities. Slow stretches (< 20°/s) were intended to measure the passive stiffness in the absence of a stretch reflex. Reflex-mediated stiffness, on the other hand, is measured by moving the joint with a sufficiently high velocity (> 300°/s) to trigger a stretch reflex in the wrist flexors [32, 72]. Throughout the assessment, the forearm of the patient was firmly fixed by the experimenter to ensure that the applied stretching force was translated into a rotational torque at the level of the wrist joint alone.

EMG signal was sampled with a frequency of 1 kHz from Flexor Carpi Radialis (FCR) and Extensor Carpi Radialis (ECR) muscles using bipolar surface adhesive electrodes (Covidien-Kendall, 24 mm) placed over the belly of the corresponding muscles with an inter-electrode distance of 2 cm (Fig. 1A). The ground electrode was placed on the ulnar styloid process. Before the placement of the EMG electrodes, the skin was prepared using abrasive gel to improve the contact with the electrodes.

The PSAD was wirelessly connected to a data acquisition software which provided online visual feedback on the velocity of the stretch and EMG activity in FCR and ECR muscles (Fig. 1B). Patients were encouraged to relax completely and to avoid actively helping or resisting the movement. When visible EMG activity was detected before the stretch or during slow stretches, the measurement was rejected and repeated after encouraging and helping the patient to relax. Each measurement session was composed of six slow and six fast stretches. All acquired data including position, acceleration, angular velocity, EMG data and forces were saved for offline analysis.

c. Electrophysiological assessment of the spinal circuitry

To measure the excitability of the post-activation depression, reciprocal inhibition and presynaptic inhibition, patients sat in an electronically-adjustable chair with their back upright, head straight and eyes open. The assessed arm was supported using a height-adjustable table. The shoulder was relaxed in a position of ~ 45° abduction and forward flexion, elbow supported by the table and slightly flexed and the forearm pronated. Since the electrophysiological measurement immediately followed the PSAD measurement, the same EMG electrodes were used to record the activity in FCR and ECR muscles. Bittium NeurOne hardware and software (Bittium NeurOne Tesla, Kuopio, Finland) were used to sample EMG data at a frequency of 10 kHz. A Tesla low pass filter, integrated within the device, was utilized with a lower cutoff frequency of 1500 Hz.

The Hoffmann reflex (H reflex) in the upper limb was measured in the FCR by stimulating the median nerve at the elbow [73]. Nerve stimulation was delivered through rectangular shocks of 1 ms duration using metal electrodes (brass) (1.5 cm radius), which were covered by saline-soaked sponges and placed over the median nerve in the cubital fossa. The optimal placement of the stimulation electrodes was ensured by checking the H and M wave responses in the EMG. To qualify as an H reflex, the response had to appear at a latency of 16–21 ms [74] and to decrease after reaching its peak amplitude when the stimulus intensity was further increased. During the paired-pulse paradigms, the radial nerve was stimulated at the spiral groove ~ 10 cm above the radial epicondyle using identical metal electrodes fixed by Velcro straps. A customized Matlab program was used to trigger two direct current stimulators (DS07 Digitimer, United Kingdom) according to the desired stimulation paradigm.

### Recruitment curve and intensity selection

**Fig. 2:**
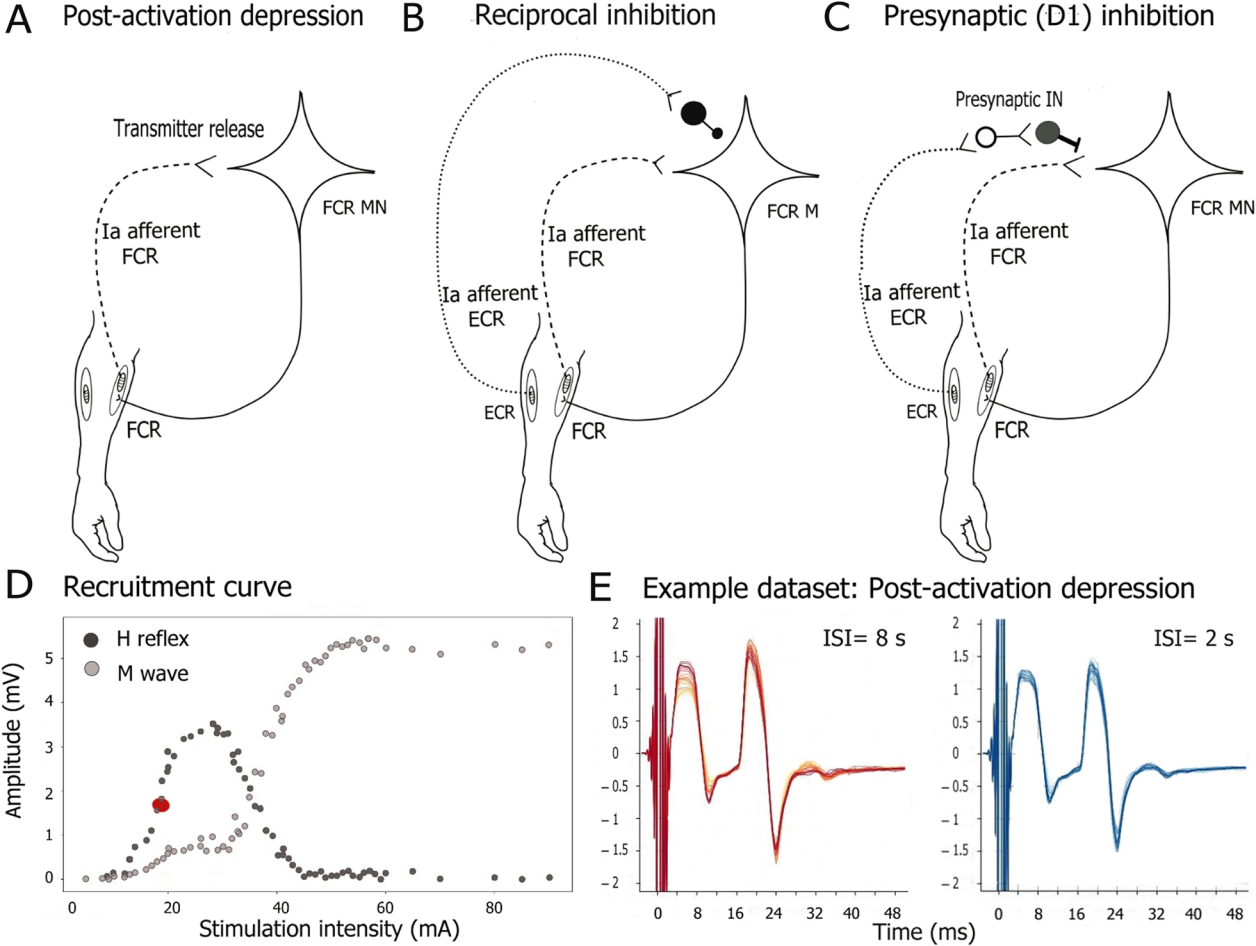
Illustration of the spinal circuit mechanisms investigated in this study. The excitability of the monosynaptic Ia afferent-motoneuron (MN) of the flexor carpi radialis muscle (FCR) is modulated by the activity in many spinal circuits. **A** Post activation depression of previously activated synapses plays a role in modification of synaptic transmission at the Ia–MN synapse and is probably related to the readily releasable neurotransmitters. **B** Reciprocal inhibition of the antagonist muscle group (extensor carpi radialis muscle (ECR)) on the wrist flexors is mediated by inhibitory interneurons which are activated by the ECR Ia afferents. **C** Presynaptic inhibition (D1) inhibition modulates the release of transmitters from Ia-MN presynaptically. **D** H reflex and M wave recruitment curve example. The amplitude of the H reflexes (dark gray circles) and the direct muscle responses (M waves: light gray circles) are plotted against the stimulation intensity in milliampere (mA). The intensity of stimulation during the experiments was set to produce an H reflex of the amplitude Hmax/2 (red circle). The maximum muscle response (Mmax) is the amplitude recorded when the M wave amplitude plateaus with increased stimulation intensity. **E** Example EMG responses to stimulation with different interstimulus intervals (ISI) during post-activation depression measurement. The red overlaid M waves and H reflexes were recorded at an ISI of 8 s, while the blue traces were recorded at an ISI of 2 s

To determine the optimal stimulation intensity, we stimulated the median nerve with varying stimulus intensities ranging between 5 and 90 mA presented in a random order (2 repetitions per intensity, total stimulations = 68) with an interstimulus interval (ISI) of 6 s. The peak-to-peak amplitudes of H reflexes and M waves were calculated for each intensity and the recruitment curve plotted (Fig. 2D). The desired stimulation intensity was adjusted to produce an H reflex with a peak-to-peak amplitude of Hmax/2 (Fig. 2D). Care was taken to select an intensity with an H reflex on the ascending phase of the input–output (recruitment) curve. Supramaximal stimulus intensity was then used to record the maximum M response (Mmax).

### Post-activation depression

Post-activation depression was measured by varying the inter-stimulus interval (ISI) of consecutive stimuli. The median nerve was stimulated with two different ISIs: 2 and 8 s (Fig. 2A). Blocks of trains of stimuli (10 stimuli per block), with an ISI of 8 or 2 s alternated until 30 reflexes at each interval were obtained (3 blocks of 10 stimuli per ISI). These stimulation blocks were intercepted by 3–4 stimuli with an ISI of 6 s, used to obtain unconditioned H reflexes of the amplitude H max/2. The outcome measure of this assessment is the calculated ratio of the H reflex amplitude evoked every 2 s to that of the H reflex evoked every 8 s: (H2/H8) (Fig. 2E).

### Inhibition of the forearm flexor motoneurons by the antagonist extensor muscle afferents: reciprocal inhibition (group I disynaptic inhibition) and presynaptic (D1) inhibition

A single electrical stimulus at motor threshold to the radial nerve at the spiral groove produces three phases of inhibition in the wrist flexor H reflex. The first occurs at a conditioning-test interval of − 1 to + 3 ms and represents the disynaptic reciprocal inhibition of flexor motoneurons by afferent input from the antagonist extensor muscle (Fig. 2B) [75]. The second phase also called D1 [76] occurs at a conditioning test interval of 5–40 ms and is produced by large diameter group I extensor muscle afferents which act on the H reflex pathway at a premotorneuronal site [54] (Fig. 2C). The third phase (D2) is much longer lasting (50–1000 ms) and is postulated to be transcortical [77].

The mechanism of the D1 inhibition which modulates the release of transmitters from Ia-MN presynaptically, was classically thought to be mediated by GABA_A_ receptor-induced depolarization of the afferent terminals (PAD) with a subsequent reduction of transmitter release [78]. However, recent studies have profoundly reshaped this interpretation. GABA_A_ receptors are predominantly located on the internodes of primary afferents, generating a “primary afferent depolarization” that secures the propagation of the action potential, reducing the likelihood of propagation failure. Consequently, synaptic transmission to the motoneurons is actually amplified rather than diminished [79, 80]. Conversely, at the Ia afferent terminals, GABA_B_ receptors (G-protein-coupled receptors) are present, which inactivate voltage-gated Ca2 + channels, resulting in a reduction of transmitter release, i.e., presynaptic inhibition [79]. These recent findings are likely to have implications for the electrophysiological measurement of presynaptic inhibition. In this study, however, we replicated the experimental protocol established by Lamy et al. [53].

The degree of attenuation of the H reflex in the FCR after stimulation of the Ia afferents in the antagonist muscle (ECR) was measured with a double stimulation paradigm. The median and radial nerves were stimulated with an ISI of 0 ms for reciprocal inhibition. The placement of the radial nerve stimulation electrodes ~ 10 cm proximal to those stimulating the median nerve allows the activity of the radial Ia afferents to reach the spinal cord 1 ms before that of the median Ia afferents. This 1 ms advanced arrival enables the radial nerve to condition the response of the FCR MN by activating an inhibitory interneuron mono-synaptically (synaptic delay ~ 1 ms). For measuring the presynaptic inhibition, the conditioning pulse to the radial nerve preceded the test pulse by 13 ms. The radial nerve was stimulated with an intensity that was at or just below the ECR motor threshold. Single stimuli of the median nerve (unconditioned H reflexes) were randomly alternated with double stimuli (conditioned H reflexes) every 6 s until 30 stimuli of each condition were collected. The outcome measure of this assessment was the amplitude of the conditioned H reflex expressed as percentage of its unconditioned amplitude.

### Signal processing and data analysis

#### PSAD data processing and extraction of passive stiffness and reflex-mediated parameters

A customized MATLAB code was used to analyze the raw data based on the methods initially described in Yamaguchi et al. [34] and modified for the wrist joint by Mahmoud et al. [38]. The amplitude of the passive stiffness, i.e., stiffness of the muscle and soft tissues in the absence of a stretch reflex was extracted from the torque data collected during slow stretches (Fig. 1B). Passive stiffness was calculated as the change in torque required to move the joint 1 degree from particular points in the range of motion (ROM), namely 30, 40, and 50% of the ROM. We averaged the stiffness values over a range that spans 5 degrees below to 5 degrees above each of the selected points (25–35%, 35–45%, 45–55% ROM). The three resulting values were then averaged to obtain one representative measure of passive stiffness per stretch. In addition, an exponential function was fitted to the slow stretch torque data to enable the estimation of the passive muscle stiffness at any given part of the ROM.

To calculate the stretch reflex-mediated torque, we identified stretch reflexes in the EMG data collected during fast stretches (Fig. 1B). The EMG signal was rectified, smoothed and filtered using a third order Butterworth band pass filter (limits: 75–175 Hz). A peak detection function was used to identify peaks in a window of 100 ms following the point of maximal velocity in the smoothed EMG data. The torque in the time interval 0–100 ms after the EMG peak was then averaged to produce a total torque value which included both active and passive components. To extract the reflex-mediated torque, we subtracted from the total torque the value of the torque during the slow stretch that was measured (or estimated using the exponential function) at an equivalent point in the ROM where the total torque was calculated i.e., 100 ms after the EMG peak.

Values representing passive stiffness and reflex-mediated torque extracted from 6 slow and 6 fast stretches in each session were then averaged to produce a single representative value per outcome per session.

#### EMG data processing and extraction of H reflex data

EMG data was imported from NeurOne and analyzed using a customized program written in Python (version 3.7.4). A Butterworth bandpass filter with a frequency range of 0.1 to 1 kHz was applied to the EMG signal.

For each stimulus, a time epoch of 150 ms was extracted (− 100 to + 50 ms, where time 0 is the stimulus) from the recorded FCR EMG data. The EMG signal in the 100 ms preceding the stimulus was visually inspected for muscle activity. The H reflexes that were preceded by muscle activity were excluded from the analysis. At least 20 reflexes per condition had to be available for averaging, otherwise the dataset for that measurement was excluded from further analysis.

In order to calculate the amplitude of the H reflexes and M waves, the program searched for positive and negative peaks within a time window that was optimized for each subject (H reflex latency varies slightly according to height, age, electrode positioning and impedance. For most subjects, however, this window was set to 18–35 ms for the H reflex and 4–13 ms for the M wave. The stability of the H reflexes was carefully examined by averaging individual responses to create a collective EMG trace and comparing the individual traces to the averaged trace. Responses which deviated in shape, width or peak latency (beyond ± 2 ms) from the average trace were excluded from further analysis. The peak-to-peak amplitudes of the individual H reflexes and M waves were then calculated and the conditioned and unconditioned responses averaged and saved for statistical analysis.

### Statistical analysis

#### Testing the effect of the intervention on the clinical scores, spasticity and the excitability of the spinal circuits

To test the change in the parameters of interest after the rTMS and physiotherapy intervention, we used multilevel analyses (linear mixed models) because of their robustness against missing data, and the ability to account for inter-subject variability by modeling it as a random effect. The method used for parameter estimation was maximum likelihood while the covariance matrix used for the repeated variable was set to be compound symmetry. SESSION was set as the repeated variable with two levels: pre and post. To correct for multiple testing, we applied Benjamini–Hochberg procedure to all the p values with a false discovery rate (FDR) of 5%.

When examining the effect of the intervention on the spinal circuit excitability, we first established the intra-subject stability of the amplitude of the unconditioned H reflex expressed as a percentage of Mmax across the two assessment sessions. To do so, we ran a one-way repeated measures analysis of variance (ANOVA) with SESSION (pre vs. post) as a repeated variable. This is important because the amplitude of the unconditioned H reflex was adjusted to Hmax/2. The analysis showed no effect of SESSION on the amplitude of the unconditioned H reflex/Mmax. It was thus considered safe to run further analyses comparing the amplitudes of the conditioned H reflexes between the two sessions. And to account for inter-subject variability in the amplitude of the unconditioned H reflex/Mmax when testing the effect of the intervention, we ran a linear mixed effects model analysis starting with the simplest model, which consisted of a single fixed repeated effect: SESSION (with two levels: pre and post). Then, the complexity of the model was increased gradually by including the amplitude of the unconditioned H reflex as a fixed variable, then as a random variable with different intercepts for each individual. The model fit was assessed using -2 Log Likelihood and the results of the best fitting model are reported.

#### The correlation between intervention-related change (post–pre) of different parameters

To explore whether change in the primary clinical outcome measure and/or stretch reflex torque were related to the degree of change in the excitability of any of the spinal mechanisms, we calculated the change (Δ) in FMA-UE, stretch reflex torque and the excitability of the three mechanisms (value post intervention–value before intervention). We then ran a non-parametric (Spearman) correlation analysis on the calculated change values. A non-parametric correlation analysis was chosen because of its robustness against outliers as well as the inability to assume linearity in the relationship between the amount of change (post–pre) of different parameters.

## Results

### Patients

Table 1 shows the characteristics of all the patients recruited for the study. Thirty-four patients had left, and 20 patients had right chronic hemiparesis. On average, time-since-stroke was 46 ± 42 months. Our patient group was heterogeneous in terms of impairment and motor function with an FMA-UE score (mean ± SD) of 26 ± 17 (range, 4–59). The MAS also varied with an average total MAS of 12 ± 7.3/70 (range, 1–35), and an average MAS wrist extension of 1.5 ± 1.3 (range, 0–4). The patient’s own impression about how much spasticity they experienced, using the visual analogue scale had a mean score of 45 ± 26% (range, 0–100), while the motor activity log (MAL), which describes the use of the affected upper limb in performing activities of daily living showed that most subjects relied almost entirely on their non-affected side for most of the activities of daily living with a MAL score of 11 ± 16% (range, 0–69). Initially, 54 patients were enrolled in the study but only 51 patients completed both pre- and post-intervention sessions.

Considering the variability in characteristics among stroke patients in our study sample, we conducted additional subgroup analyses to investigate how these patient characteristics might have influenced the main findings. These analyses can be found in Additional file 1.

### Effect of the intervention on the clinical scores

The mean values and standard deviations of the clinical scores before and after the intervention, as well as the results of the statistical analysis for each outcome parameter are listed in Table 2. The analysis showed a significant improvement in the FMA-UE: (F (1,51.1) = 36.3, p < 0.001), as well as in the WMFT, both in the time (F (1,26.2) = 9.8, p = 0.004) and function (F (1,26.1) = 12.2, p = 0.002) components, but not the grip strength (F (1,25.1) = 0.088, p = 0.77). Note that only those patients with sufficient residual arm ability underwent the WMFT in order to avoid causing frustration for those patients who are unable to initiate any of the functional tasks which constitute the WMFT.

**Table 2:** Intervention-related change in the clinical scores

Clinical outcome	N	Mean ± SD Pre	Mean ± SD Post	F	p
FMA-UE/66	54	25.9 ± 16.9	29.9 ± 18.4	36.3	**< 0.001**
WMFT-time (s)	30	878.5 ± 473	721 ± 441	9.8	**0.004**
WMFT-function/75	30	34.9 ± 18.5	40.6 ± 19.3	12.2	**0.002**
WMFT-grip strength (kg)	30	10.7 ± 7.2	11.8 ± 7.0	0.09	0.77
MAS wrist extension (0–4)	54	1.47 ± 1.1	1.25 ± 1.0	10	**0.003**
MAS arm total/70	53	12.1 ± 7.3	10.4 ± 6.8	10.3	**0.002**
Visual analogue scale (%)	52	45.1 ± 26.1	44.3 ± 28.9	0.00	0.99
Motor activity log (%)	50	12.1 ± 16.2	17.0 ± 22.2	8.6	**0.005**
Disability rating scale/24	53	12.3 ± 4.8	12.2 ± 5.6	0.035	0.851

N: number of subjects; FMA-UE: Fugl Meyer Assessment-Upper extremity; WMFT: Wolf Motor Function test; MAS: modified Ashworth scale. Significant p-values after correction using False Discovery Rate (FDR) Benjamini–Hochberg are indicated in bold

Spasticity estimated using the modified Ashworth scale (MAS) was significantly reduced, both when examining the wrist extension movement alone (F (1,49.9) = 10, p = 0.003) or when considering the total score which includes multiple arm joints and movements (F (1,50.3) = 10.33, p = 0.002).

The analysis showed no significant change in the visual analogue scale of spasticity or the disability rating scale after the intervention. On the other hand, the MAL, a more robust measure of the use of the affected upper limb in everyday life, showed a statistically significant response to the intervention with an almost 5% average increased use of the affected upper limb in activities of daily living.

### Effects of the intervention on reflex-mediated stiffness (spasticity) and passive stiffness components of resistance to wrist stretch

**Fig. 3:**
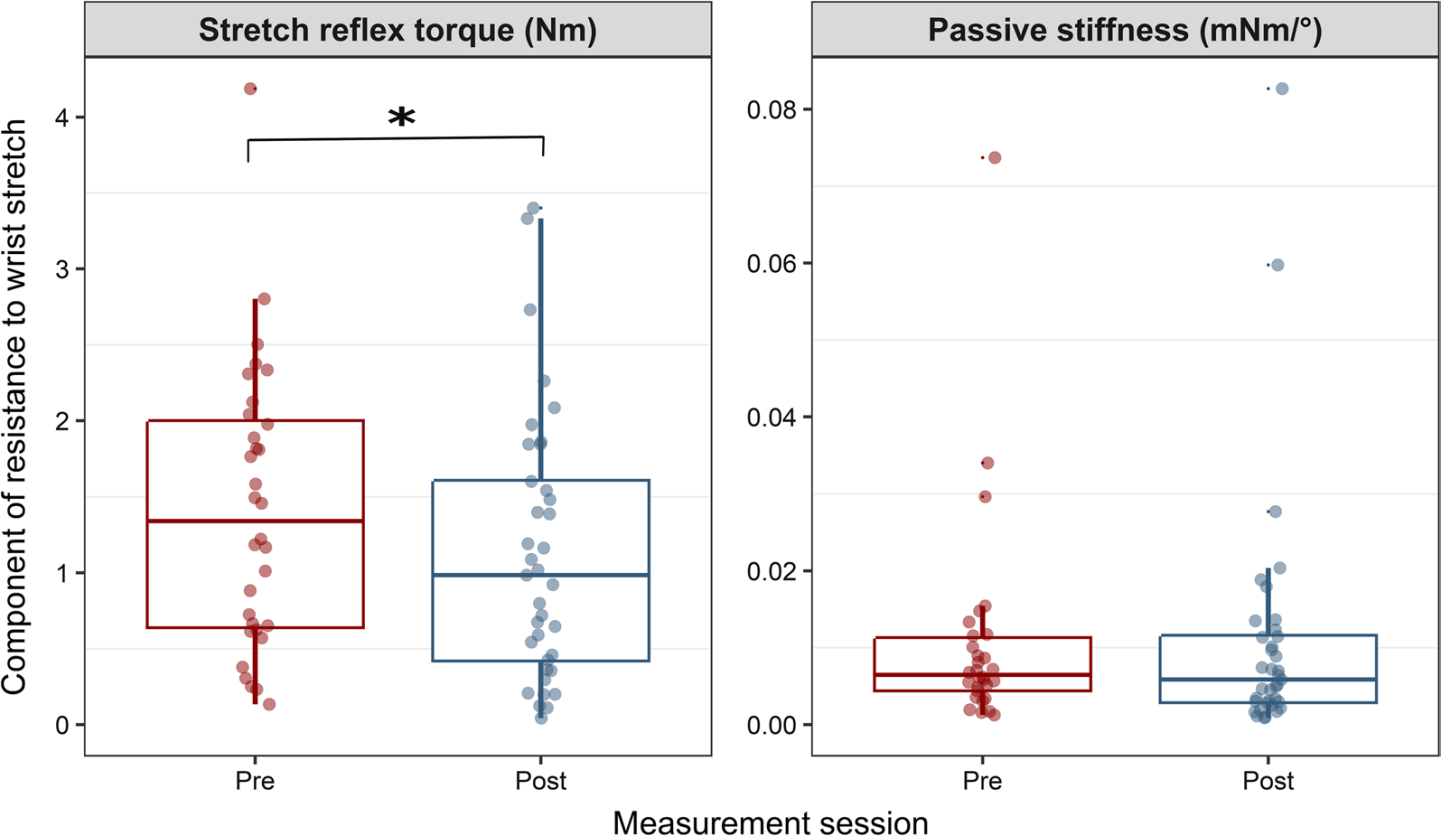
Boxplot which displays the amplitude and distribution of the parameters measured using the PSAD device prior to (Pre) and after the rTMS intervention (Post). The middle horizontal line of each box represents the median value. The lower horizontal lines represent the first quartile (Q1: 25th percentile) while the upper horizontal line represents the third quartile (Q3: 75th percentile). The whiskers indicate the spread of data points smaller than the first or larger than the third quartiles (in the range of 1.5 × interquartile range (IQR). The red and blue circles represent individual data points pre and post intervention, respectively. The stretch reflex torque is the torque measured in a 100 ms window that follows the maximum EMG peak around maximum velocity. Passive stiffness is the component measured during slow stretches in the absence of stretch reflex, it represents changes in the elasticity of muscle and soft tissue due to immobilization. Nm: Newton-meter; mNm/°: milli Newton-meter per degree. The asterisk (*) indicates a significant difference at the level p < 0.05

The results of the linear mixed model analysis showed that the intervention had an exclusive effect on the active component of resistance to passive stretch measured using the hand-held dynamometer (Fig. 3). The stretch reflex-mediated torque was significantly reduced after the intervention *F* (1,32.5) = 5.7, *p* = 0.023 while the passive stiffness component did not change *F* (1,31.6) = 0.5, *p* = 0.49.

### Effects of intervention on the excitability of the spinal circuitry

**Fig. 4:**
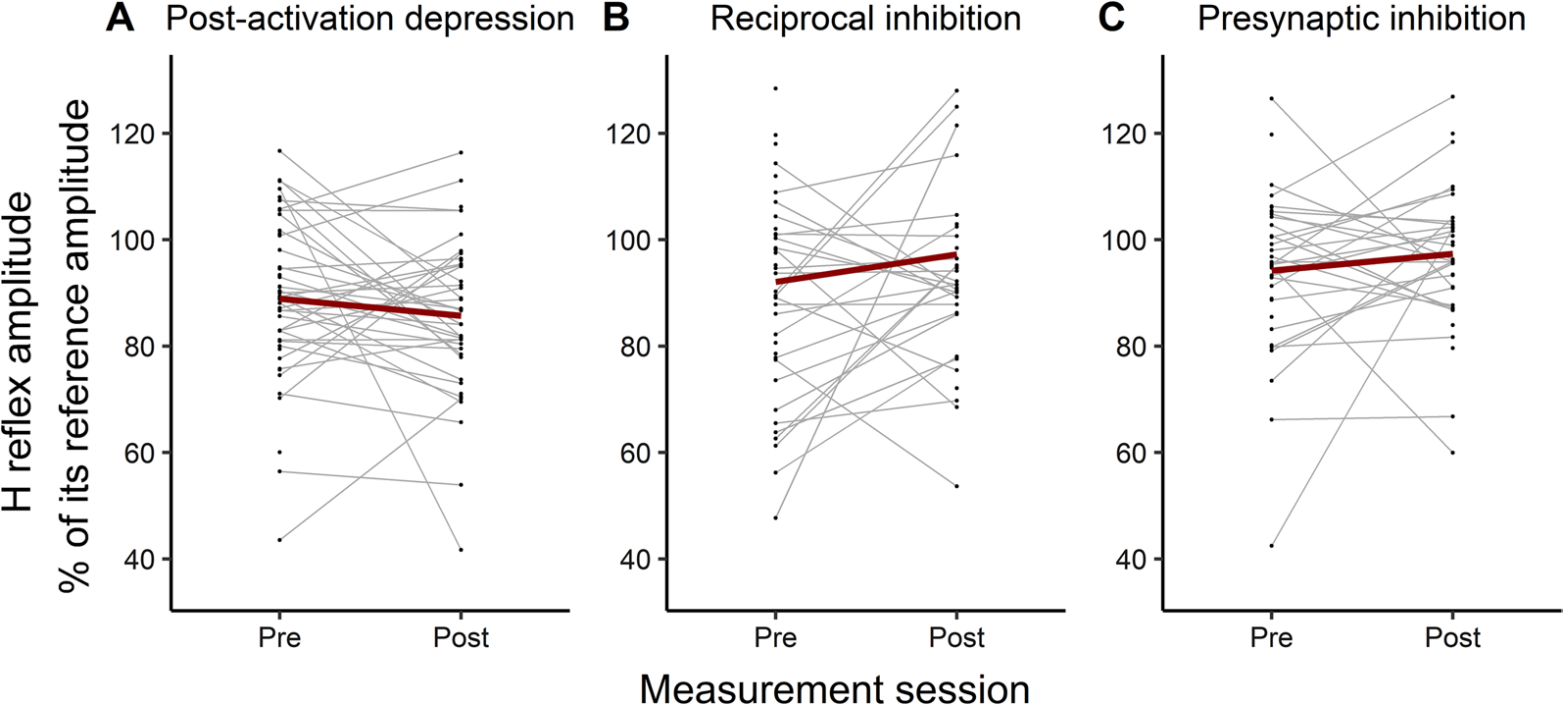
Excitability of the three spinal circuit mechanisms **A** post-activation depression; **B** reciprocal inhibition; **C** presynaptic inhibition) pre and post intervention. Each line represents one subject and connects the pre and post values (black dots) of that subject. The thick red lines represent the mean values for all subjects for each mechanism

In all three analyses (post-activation depression, reciprocal inhibition and presynaptic inhibition), the model with the best fit was the simplest with a single fixed repeated effect: SESSION (with two levels: pre and post). Modeling the amplitude of the unconditioned H reflex as a random variable in the linear mixed effects model did not improve its fit. The analysis showed no effect of the intervention on the amount of the post-activation depression (F (1,46.39) = 1.67, *p* = 0.20), presynaptic inhibition (F (1,39.9) = 1.08, *p* = 0.30), or reciprocal inhibition (F (1,43.93) = 2.4, *p* = 0.13) (Fig. 4A–C).

### The correlation between the extent of change in clinical outcomes and spasticity with change in the excitability of the spinal circuit mechanisms

**Fig. 5:**
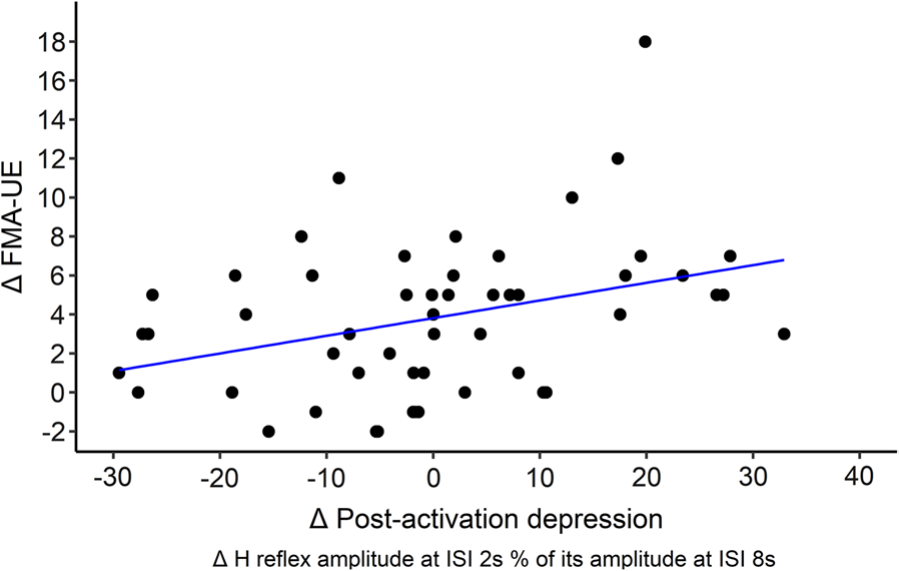
Correlation between post minus pre intervention change (Δ) in FMA-UE (with positive values indicating clinical improvement) and change (Δ) in the amplitude of post-activation depression. Note that post-activation depression is expressed as a ratio of the amplitude of H reflex measured every 2 s to that measured every 8 s, with positive post minus pre intervention values indicating reduction of post-activation depression

Table 3 shows the results of the Spearman correlation analyses between post minus pre intervention changes in the FMA-UE and the stretch reflex torque with post minus pre intervention changes in the excitability of the spinal mechanisms. Only the change in FMA-UE score and the change in post-activation depression were significantly correlated. This correlation indicated that patients who had a greater increase in the FMA-UE score had a greater decrease in post-activation depression (Fig. 5).

**Table 3:** Correlation between the post minus pre interventional changes of FMA-UE and stretch reflex torque with those of the excitability of the spinal mechanisms

Correlated parameters (change)	Correlation coefficient	p
FMA-UE—Post-activation depression	0.41	**.002**
FMA-UE—Reciprocal inhibition	0.09	0.58
FMA-UE—Presynaptic inhibition	− 0.03	0.86
Stretch reflex torque—Post-activation depression	− 0.09	0.62
Stretch reflex torque—Reciprocal inhibition	− 0.20	0.33
Stretch reflex torque—Presynaptic inhibition	− 0.05	0.79

## Discussion

The investigation of the effect of rTMS and physiotherapy intervention on spasticity and the associated spinal circuitry revealed the following: (1) The intervention resulted in a significant reduction in motor impairment, an improvement in motor function and a reduction in the clinical measures of spasticity; (2) There was a significant reduction in spasticity measured objectively using a hand-held dynamometer. This reduction was exclusive to the active component of the response to passive wrist stretch measured as stretch reflex torque, while no change was recorded in the passive stiffness component; (3) The reduction in spasticity was not accompanied by change in the excitability of post-activation depression, reciprocal inhibition or presynaptic inhibition; (4) A significant correlation was found between the reduction in motor impairment (increase in FMA-UE) and the reduction in post-activation depression.

### Positive effects of rTMS and physiotherapy intervention on clinical scores of motor impairment and function

The application of low-frequency rTMS over the motor cortex in the contralesional hemisphere decreases the excitability of the stimulated hemisphere, which leads to an indirect increase in the excitability of the non-stimulated ipsilesional hemisphere by reducing the interhemispheric inhibition from the stimulated to the non-stimulated motor cortex [81–84]. This is the mechanism thought to underlie rTMS-induced neuroplastic changes used to promote the efficiency of rehabilitation after stroke [85–87]. In this study, our patient population experienced a significant reduction in motor disability and an improvement in motor function evident by a significant increase in both the FMA-UE and WMFT scores. The increase in the WMFT scores included both the “time” and “function” components, which is important, because the functional score of the WMFT describes the quality of the movement including the fluidity and the influence of synergy on its performance.

The improvement in motor function was reflected in an increased use of the affected hand and arm in activities of daily living, measured using the MAL. This is probably the most important aspect of any rehabilitation intervention, as the increased use of the arm indicates that the intervention-related changes are clinically meaningful. Moreover, the ability to apply the arm and hand in everyday life facilitates the continuation of the rehabilitative process after the end of the therapy as a high repetition of exercise movements is necessary for motor improvement [88].

### RTMS and physiotherapy intervention has an exclusive effect on the stretch reflex torque

There is accumulating literature that indicates a positive effect of low-frequency rTMS on spasticity evaluated using the MAS for reviews, see [19, 89–91]. The results of our clinical data analysis also showed a significant reduction in the total MAS score and the MAS score for wrist extension. These findings add little to our understanding of the pathophysiology of spasticity and the possible effects that different interventions may have on it. This is due to significant mismatches between the clinical scale and the definition of spasticity. First, the “velocity dependency” aspect of the response to the stretch cannot be ascertained using the MAS. During the performance of the test, the examiner moves the joint and simultaneously estimates the perceived resistance without precisely controlling the velocity of the movement. Second, the “stretch reflex” component of resistance to joint stretch is almost certainly indistinguishable by the clinician from increased resistance caused by shortening of the muscle–tendon complex and changes in their microstructure, i.e., muscle contracture [25–29].

To the best of our knowledge, this is the first study to explore the effect of low-frequency rTMS and physiotherapy intervention on spasticity measured objectively using a device which enables the discrimination between the reflex-mediated and passive stiffness components of the response to muscle stretch. Our findings indicated that the intervention had an exclusive effect on the stretch reflex torque (Fig. 3), confirming that contralesional low-frequency rTMS indeed has a positive effect on reducing spasticity in chronic stroke.

The outcome that the passive resistance to wrist stretch remains unaffected by the intervention, aligns with expectations, considering that rTMS primarily influences the neurogenic component of resistance. This occurs through alterations in the excitability of corticospinal neurons, which in turn affect spinal interneurons and modulate their activity [18, 61, 63, 92, 93]. Given the novelty of the application of the objective measurement tool in chronic stroke, and the limitation of the study design, these results need to be replicated in a randomized sham-controlled paradigm.

### No intervention-related changes in post-activation depression, reciprocal inhibition and presynaptic inhibition

We expected that a reduction in spasticity would be accompanied by changes in the spinal mechanisms, which seem most likely related to the development of spasticity. Indeed, previous studies demonstrated that rTMS can change the excitability of the spinal circuitry [16–18, 92]. For example, Perez et al. (2005) found that a short train of high-frequency rTMS (20 pulses, at an intensity of 1.2 × MEP threshold and a frequency of 5 Hz) over the leg motor cortical area in healthy subjects increased presynaptic inhibition of Ia afferents which project onto soleus motoneurons. Meunier and Pierrot-Deseilligny (1998) also reported that TMS provided an increase in the radial-induced inhibition of the wrist flexor H-reflex which suggests an increase in presynaptic inhibition. In our findings, none of the examined spinal mechanisms showed a significant change after the intervention.

There are multiple possible explanations for this finding, the first is related to the time scale of the effect of rTMS on the spinal circuits. Previous studies compared the activity before and after short interventions with a maximum intervention-measurement delay of 30 min [16–18, 94]. The effects of rTMS on the spinal circuits may be short-lived as reported by Perez et al. [18] who found that the size of the H reflex returned to its normal size at a stimulus-test interval of 3 s. It is important to consider that in the aforementioned studies, the stimulation intervention was also short, unlike the multi-session rehabilitative intervention that our patients underwent. It is possible that short-term effects on the spinal circuits themselves trigger secondary, and longer lasting changes in other mechanisms related to the firing threshold of motoneurons [95, 96].

Another possibility concerns the actual relevance of the examined spinal mechanisms to the pathophysiology of spasticity. There seems to be agreement in the literature that the reduction in post-activation depression is at least one of the primary mechanisms underlying spasticity [97–99]. It has been consistently found to be reduced on the affected but not the unaffected side in spastic patients after stroke [50–53] and to be related to the degree of spasticity measured with the Ashworth scale in the upper limb [53]. Substantial evidence is also available for the reduction of reciprocal and presynaptic inhibition in the upper limb in the stroke population [54–56], but the contribution of these mechanisms to the development of spasticity is uncertain [44, 46, 53]. It is not unlikely that intervention-related changes might have taken place in other spinal circuits, ones that we have not measured. A likely candidate is the one that underlies the inhibition in the third phase of radially induced FCR inhibition. This inhibition, with a latency of 100 to 300 ms, is caused by long-loop inhibitory connections to supraspinal centers that receive input from the premotor cortex. In a similar study, in healthy subjects, Huang et al. [16] found that continuous theta-burst stimulation had no effect on the first and second phases of inhibition between forearm extensor and flexor muscles. However, they did find a reduction in the amount of inhibition in the third phase. Similar findings were reported by Huang et al. [17] in patients with dystonia. They suggested that if the third phase of radially induced inhibition of FCR is due to activity in a long-loop spinal– brainstem–spinal pathway, then the effect on this phase may be due to changes in activity of premotor-brainstem connections.

One must also keep in mind that the inherent variability in the H reflex measures may have masked underlying changes, a concern that was considered by Nielsen et al. [46] in their review, as an obstacle in the way of progressing our understanding of the pathophysiology of spasticity. Even within the healthy population, disynaptic reciprocal inhibition, for example, was found to be absent in many subjects, although they are not spastic [100]. H reflex measures are even more challenging in the upper limb than in the lower limb, with a large cross-talk between the flexor and extensor EMG channels due to their physical proximity. In addition to the variability in the physiological measurements themselves, our patients represent a heterogeneous population with different lesion sizes and locations. The subsequent adaptations in the spinal networks as a response to the primary lesion may vary considerably between the individuals, and so may the adaptation in these networks to the intervention.

### The excitability of spinal mechanisms might be related to motor recovery

A noteworthy observation arises when examining the association between changes in motor disability and the excitability of spinal circuitry. While the significant correlation between the reduction in motor impairment and post-activation depression following intervention (Fig. 5) does not establish causality, it prompts further investigation into the underlying physiological mechanisms driving motor recovery.

After an injury to the central nervous system, multiple mechanisms are triggered, which aim to amplify the signal arriving at the motoneuron from the residual intact pathways [41]. Examples of these processes include collateral sprouting and the formation of new synapses from intact axons onto partially denervated motoneurons [101–104] systematic changes in gene expression and the upregulation of the essential transmitter receptors [105]; and the downregulation of the potassium-chloride cotransporter [106]. All of these factors contribute to an increased excitability below the lesion (thus certainly contributing to the active stretch reflexes in spasticity), but they may also facilitate the recovery of voluntary movement (with specific motor patterns) even when the descending pathways mediating this information are weakened by the lesion.

Indeed, motor recovery and the development and ultimate disappearance of spasticity are interconnected and go through different stages during post stroke complete recovery [43, 107–109]. The direction (facilitation vs. inhibition) in which the excitability of a certain spinal circuit or mechanism needs to develop in order to allow movement to take place probably depends on many factors. It is perhaps naive to assume that increased excitability of the corticospinal tract as a result of the rTMS intervention should translate into global reduction in the hyperexcitability of the involved spinal circuitry. In order to enable motor recovery during the rehabilitative process, it is plausible that the nervous system might still need to facilitate, even further, some of the processes which may amplify the descending corticospinal input.

Whether spasticity per se needs to be considered a target for therapeutic interventions is, to say the least, disputable [110–114]. There is evidence that mechanisms other than spasticity underlie the spastic movement disorder including the impaired ability to voluntarily activate muscles, i.e., paresis, muscle atrophy [115] and co-activation of antagonist muscles [113, 114, 116–118]. Nonetheless, interventions targeted towards motor rehabilitation, including motor cortex rTMS, which simultaneously improve motor function and reduce spasticity by improving motor control and (probably) increasing ipsilesional corticospinal tract excitability [81–84] might mimic natural recovery, in which spasticity does decrease as a function of improvement in motor control [107] and could eventually disappear in the case of full recovery [109].

## Limitations

One limitation of this study is the absence of an age-matched control group, potentially limiting the generalizability of our results. However, it is essential to highlight that our study builds upon established randomized sham-controlled trials, which have demonstrated the efficacy of rTMS interventions on motor impairment and function [86, 119–122], and on spasticity [6–10, 91] in chronic stroke. Our clinical findings both in relation to the motor function and to the MAS align with these previous studies.

Our primary objective was not to prove the effectiveness of the intervention but to delve into its underlying mechanism. We used a novel technology to carefully measure and distinguish the reflex-mediated and passive stiffness components of the response to muscle stretch and their responsiveness to the intervention. To the best of our knowledge, this is the first study to combine clinical, biomechanical, EMG and electrophysiological measurements to explore the impact of low-frequency rTMS and physiotherapy on spasticity. This was made possible by recent development of clinically applicable measurement devices and their demonstrated reliability in this population [34, 38]. Additionally, we aimed to investigate the spinal circuitry mechanisms, which might underlie spasticity and their response to rTMS and physiotherapy intervention. The use of a within-subject design is legitimate for the purpose of identification of possible underlying mechanisms.

Another limitation lies in the concurrent use of rTMS and regular physiotherapy in our patient population. Both interventions have been shown to modulate recovery, even in chronic stroke [88]. Consequently, the effects observed in our study cannot be solely attributed to rTMS but rather to the combination of both interventions. Again, it is important to note that our study was not designed to establish the superiority of rTMS over physical therapy. Priming motor rehabilitation with rTMS has become a standard practice in stroke rehabilitation, with evidence indicating that the combination of rTMS with immediately subsequent upper-limb training showed better outcomes in improving motor disability and function compared to upper-limb training alone [81, 123–126].

While recognizing the need for confirming the effect of rTMS and physiotherapy intervention on stretch reflex reduction with a randomized controlled design, our initial findings serve the purpose of presenting preliminary evidence. These findings encourage further exploration of the intervention's effects in fully powered and controlled research settings.

## Conclusions

One Hz rTMS of the contralesional motor cortex in combination with physiotherapy in chronic motor stroke patients triggered a specific reduction in the stretch reflex mediated torque measured in the wrist flexors using a hand held-dynamometer. In this study, we were unable to clarify the specific mechanisms by which rTMS interacts with the spinal circuitry that is thought to contribute to spasticity. Development of new methods for more reliable assessment of spinal circuit excitability will be necessary to further our understanding of the physiological mechanisms underlying spasticity reduction.

### Supplementary Information

**Additional file 1.** Patient subgroup analyses.

## Data Availability

The datasets generated during the current study are not publicly available due to concerns regarding patient data privacy, but are available from the corresponding author on reasonable request.

## Abbreviations

APB: Abductor pollicis brevis
ECR: Extensor Carpi Radialis
EMG: Electromyography
FCR: Flexor Carpi Radialis
FDI: First dorsal interosseous
FDR: False discovery rate
GABAA: Gamma-Aminobutyric Acid Type A
GABAB: Gamma-Aminobutyric Acid Type B
Hz: Hertz
ISI: Interstimulus interval
IQR: Interquartile range
MAS: Modified Ashworth scale
MEP: Motor evoked potential
MN: Motoneurone
MRI: Magnetic resonance imaging
Mmax: Maximum M Response
N: Number of subjects
PAD: Primary afferent depolarization
POST: Post-intervention
PRE: Post-intervention
PSAD: Portable Spasticity Assessment Device
ROM: Range of motion
RMT: Resting motor threshold
WMFT: Wolf motor function test
