# Supplementary material for: Testing spasticity mechanisms in chronic stroke before and after intervention with contralesional motor cortex 1 Hz rTMS and physiotherapy
Source: J Neuroeng Rehabil. 2023 Nov 8;20:150. doi: 10.1186/s12984-023-01275-9 (PMC10631065; doi:10.1186/s12984-023-01275-9)
Supplement: Supplementary file 1 — Additional file 1. Patient subgroup analyses. [file 12984_2023_1275_MOESM1_ESM.pdf]

Additional file 1

1.1 Impact of patients without spasticity on the overall study findings

In light of the expectation that the way rTMS would interact with the physiological mechanisms underlying spasticity and the excitability of the investigated spinal circuitry would be uniform across different patients, we did not exclude patients who presented without symptoms of spasticity form the analysis. It is, however, prudent to assess how the inclusion of these patients might have influenced our overall findings.

To explore this, we created a subset of the data composed only of the subjects who were considered to have spasticity in the wrist joint according to the modified Ashworth scale (MAS) (patients who had as scores of 1, 1+, 2, 3 or 4 were included and those who scored 0 were excluded (n=13)). We then examined whether: 1) the intervention had an effect on the stretch reflex torque and passive stiffness components of resistance to passive stretch; 2) there was any change in the excitability of the investigated spinal circuits.

Our statistical approach remained consistent with the primary analysis, namely multilevel analyses via linear mixed models, with INTERVENTION as the repeated variable (2 levels: pre, post). We employed maximum likelihood for parameter estimation, with a compound symmetry covariance matrix for the repeated variable.

Results from the linear mixed model analysis for the subgroup of patients with MAS-defined spasticity closely resembled those observed in the overall study population. The intervention had a significant effect on the active component of resistance to passive stretch, as measured by the hand-held dynamometer. The stretch reflex-mediated torque was significantly reduced following the intervention ($F(1, 49.6) = 6.13$, $p = 0.017$), while the passive stiffness component remained unchanged ($F(1, 31.6) = 0.5$, $p = 0.49$). In line with our expectations, patients without spasticity exhibited no change in their spasticity levels ($F(1, 7.5) = 2.56$, $p = 0.15$).

Subsequently, we examined the effect of the intervention on the excitability of post-activation depression, presynaptic inhibition and reciprocal inhibition in the subgroup of patients with spasticity, using the same statistical analysis. The results indicated, also similar to the findings in the full sample, no significant changes in any of these examined spinal circuits. In summary, we can conclude that inclusion of the subgroup of patients with MAS = 0 did not have an influence on the overall findings of the study.

1.2 Subgroup analysis

Our patient sample presented with significant heterogeneity both with regards to the time since stroke and the level of disability measured with the FMA-UE. We, thus, wanted to explore whether the response to the rTMS and physiotherapy intervention might have been different in subgroups of patients who had similar characteristics. To do so, we created subgroups of patients based on the following criteria:

Chronicity (time since stroke -in months):

06-24 : chronicity level 1
25-45 : chronicity level 2
>45 : chronicity level 3

Disability (FMA-UE Pre) :
40-60 : disability level 1
20-40 : disability level 2
<20 : disability level 3

We then performed a repeated measures ANOVA to test the effect of the INTERVENTION (2 levels: pre, post) on the stretch reflex torque. This time, CHRONICITY (3 levels: 1, 2, 3) and DISABILITY (3 levels: 1, 2, 3) were included in the model as covariates –separately. In each model, the interactions INTERVENTION*CHRONICITY and INTERVENTION*DISABILITY were explored.

The results of the analysis did not reveal any significant interaction between INTERVENTION*CHRONICITY when run over the whole sample ($F(1,28)= 3.7$, $p=0.063$), or only those identified as having spasticity ($F(1,20)=3.8$, $p=0.066$), or between INTERVENTION*DISABILITY for the whole sample ($F(1,28)= 0.077$, $p=0.78$), or only those identified as having spasticity ($F(1,20)=0.45$, $p=0.71$).

In addition, we used Pearson's correlation analysis to explore whether there is a correlation between the time since stroke or FMA-UE with the amount of change (post- pre) in stretch reflex torque. These analyses revealed no significant correlations (all p values > 0.9).

We can thus conclude that the heterogeneity of the patient sample had no impact on the primary findings of this study.
